# Identity, positionality and reflexivity: relevance and application to research paramedics

**DOI:** 10.29045/14784726.2022.09.7.2.43

**Published:** 2022-09-01

**Authors:** Caitlin Wilson, Gillian Janes, Julia Williams

**Affiliations:** University of Leeds; North West Ambulance Service NHS Trust ORCID iD: https://orcid.org/0000-0002-9854-4289; Manchester Metropolitan University ORCID iD: https://orcid.org/0000-0002-1609-5898; South East Coast Ambulance Service NHS Foundation Trust; University of Hertfordshire ORCID iD: https://orcid.org/0000-0003-0796-5465

**Keywords:** allied health personnel, clinical academic, emergency medical services, methodology, research

## Abstract

This article introduces the reader to the concepts of identity, positionality and reflexivity and outlines their relevance to research paramedics. We outline how a researcher’s identity and positionality can influence all aspects of research, including the research question, study design, data collection and data analysis. We discuss that the ‘insider’ position of paramedics conducting research with other paramedics or within their specific clinical setting has considerable benefits to participant access, understanding of data and dissemination, while highlighting the difficulties of role duality and power dynamics. While positionality is concerned with the researcher clearly stating their assumptions relating to the research topic, the research design, context and process, as well as the research participants; reflexivity involves the researcher questioning their assumptions and finding strategies to address these. The researcher must reflect upon the way the research is carried out and explain to the reader how they moved through the research processes to reach certain conclusions, with the aim of producing a trustworthy and honest account of the research. Throughout this article, we provide examples of how these concepts have been considered and applied by a research paramedic while conducting their PhD research studies within a pre-hospital setting, to illustrate how they can be applied practically.

## Introduction

Positionality and reflexivity are concepts that are discussed in great detail in research methods literature and are widely acknowledged to be an important consideration when planning and conducting research (Huberman & Miles, 2002; Savin-Baden & Major, 2013). However, in the limited body of evidence pertaining to paramedic research methods, these concepts are only briefly mentioned (Williams, 2012). We found several examples of positionality statements and reflexivity considerations in published articles and masters/doctoral theses by paramedics conducting research (Eaton-Williams et al., 2020; Horrocks, 2020; Mausz et al., 2022; Whitley, 2020; Wolff, 2019). Often these statements and discussions vary in the detail provided, with many paramedic research outputs not including any discussions around the researcher’s positionality and reflexivity. This may be due to the stringent word count limits of journal articles but could also indicate a limited understanding or lack of awareness of these complex concepts among paramedics conducting research.

Increasingly, we are seeing articles by clinician-researchers and clinical academics in nursing and the allied health professions highlighting the relevance and applicability of identity, positionality and reflexivity to their respective professions (Hiller & Vears, 2016; Hunt et al., 2011; Marshall & Edgley, 2015; McNair et al., 2008). The aim of this article is to outline the relevance of identity, positionality and reflexivity to research paramedics by offering a definition and explanation of these concepts, including illustrated examples of how they have been applied by the lead author during their PhD research studies.

## Definitions

In this article, identity refers to professional identity, which is a dynamic concept describing how an individual perceives themselves within their occupational context and how they communicate this to others (Neary, 2014). Although not clearly defined in the literature, professional identity encompasses the ability to perform profession-specific functions, have profession-specific knowledge, identify with a community of practice and act in accordance with the values and ethics of the profession (Fitzgerald, 2020).

Positionality refers to the position a researcher has chosen to adopt within a given research study (Savin-Baden & Major, 2013). It necessitates the researcher consciously examining their own identity to allow the reader to assess the effect of their personal characteristics and perspectives in relation to the study population, the topic under study and the research process.

Reflexivity is a form of critical thinking that involves addressing the issues of identity and positionality by making the researcher’s assumptions explicit and finding strategies to question these (Lazard & McAvoy, 2017). The researcher must reflect upon the way research is carried out and explain to the reader how they moved through the research processes to reach certain conclusions, with the aim of producing a more trustworthy and honest account of the research (Corlett & Mavin, 2018).

**Figure 1:**
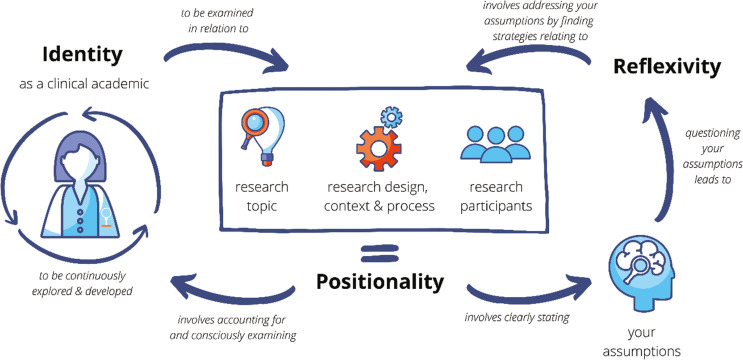
Figure 1. Identity, positionality and reflexivity for clinical academics.

The processes and connections between identity, positionality and reflexivity have been summarised visually in Figure 1.

## Research paradigms and methodologies

The concepts of positionality and reflexivity are most frequently mentioned in connection with qualitative research and particularly within the critical theories paradigm, where researchers are encouraged to be aware of their position and the way this shapes the production and interpretation of knowledge (Kincheloe & McLaren, 2008). Qualitative research involves a complex interaction between the researcher and the research subject, in which the researcher’s relationship with participants and the research process influences the findings (Pelias, 2011).

However, increasingly it is being acknowledged that researchers’ positionality plays a role across the whole range of research paradigms, including quantitative and mixed-methods research (Knoblauch, 2021; Walker et al., 2013). Jafar (2018) argues that all researchers direct their research based on countless factors and that leaving out the positionality of the research team and context deprives the reader of the opportunity to determine how important these factors might be. Within this article, we have taken the stance that identity, positionality and reflexivity are important concepts for researchers to understand regardless of which research paradigm they align themselves with; although, the specific nuances and applications may vary.

## Interactive workshop

In preparation for this article, we conducted an interactive workshop on research paramedic identity, positionality and reflexivity at the College of Paramedics Annual International Research Conference on 29 November 2021. The online workshop lasted for 45 min and was facilitated by CW and JW. The number of attendees fluctuated throughout the workshop from eight to 12, with the majority being from the United Kingdom and holding a dual role of paramedic and early career researcher. Definitions of ‘early career researcher’ vary in the literature, but we are using this term to describe individuals in the transition phase from being a post-graduate student to becoming an independent senior researcher. Workshop attendees were presented with some of the information discussed in this article and invited to share their experiences relating to identity, positionality and reflexivity.

Workshop attendees were informed of the facilitators’ intention to write a journal article on this topic and invited to share any concerns regarding this at the time or by contacting the facilitators via the provided email address. However, no concerns were raised. While writing the journal article, we have referred at times to the collective experience of the workshop attendees to aid explanation and demonstrate relevance; however, we have not included verbatim quotes or individual stories consistent with a research study and therefore did not require ethical approval, as this is a methodological discussion article.

## Identity

### The paramedic profession

The paramedic profession has undergone significant change in the United Kingdom and internationally, with many countries now offering paramedic registration and academic pathways to becoming a paramedic (McCann & Granter, 2019; Reed et al., 2019). A recent global Delphi study provided a definition of paramedicine, which acknowledged paramedics’ specialist skillset and wide range of potential working locations (Williams et al., 2021). Despite this variety, paramedics – including student paramedics – have been found to have a strong and positive image of their professional identity (Eaton et al., 2021; Johnston & Bilton, 2020; Lloyd-Jones, 2015).

The research on paramedic identity is a welcome development following a paper by O’Meara (2011) which emphasised the need for paramedics to examine their role and place in society. This establishment of a true professional identity for paramedics is thought to facilitate the development of paramedics’ own research base (Rosser, 2012), which raises questions about what we should call paramedics who conduct research.

### Research paramedics

Research is a recognised career pathway for paramedics which is increasingly gaining popularity. The concept of identity is especially important for paramedics conducting research because the identity of the researcher informs every aspect of the research process – from guiding research questions to the collection and analysis of data (Castelló et al., 2021).

However, there does not appear to be a consensus regarding the terminology used for paramedics who conduct research. Our workshop attendees from the United Kingdom utilised the term ‘research paramedic’, but this did not appear to be commonplace in other countries, where descriptive phrases such as ‘paramedic and researcher’ and ‘paramedic who does research’ were used. Depending on an individual’s job description and daily tasks, terms such as ‘paramedic research fellow’ or ‘paramedic lecturer’ were felt to provide a more accurate picture. The term ‘clinical academic’ was also used, which was believed to potentially translate better when communicating with people from a medical background or drawing parallels with clinical academics from other allied health professions.

A similar dilemma is faced by paramedics making the transition to academia, where the term ‘no man’s land of professional identity’ (Munro et al., 2018, p. 33) is used to describe paramedic academics struggling to fuse their paramedic and academic identities. The difficulties surrounding the transition experience from clinician to academic, as well as the boundary-spanning role of clinical academics, have also been described for nursing and allied health professionals (Kluijtmans et al., 2017; Murray et al., 2014). In addition to ‘clinical academic’, research-active healthcare professionals are sometimes referred to as ‘clinician-researchers’ or ‘clinician-scientists’ in the broader literature (Kluijtmans et al., 2017; Newington et al., 2022).

A personal account of the corresponding author’s difficulty in describing her identity as a paramedic and a researcher is provided in Table 1.

**Table 1.** Illustrated example – identity.

When I started my PhD, I identified both as a paramedic and a PhD student. Depending on the setting, I would introduce myself as one or the other. Nowadays, when among my ambulance service colleagues, I am most likely to use the term ‘research paramedic’, as this is a term they are familiar with and indicates to them that I am a paramedic who is involved in research. When interacting with other researchers – especially other allied health professionals who are researchers – I am increasingly using the term ‘clinical academic’. This may be due to this being a shared expression among allied health professional researchers and also a widely understood term among non-clinician researchers because of its longstanding use for medics. In the UK, this term is also a feature of the development of the clinical research infrastructure to include non-medical professionals, e.g. National Institute for Health Research clinical academic pathways. My use of different terminology depending on my immediate surroundings and target audience illustrates that I am transitioning and balancing identities. However, it also shows that my dual identity has developed over time, as I am now increasingly using terms that encompass both of these identities (research paramedic, clinical academic), rather than separate ones (paramedic and/or PhD student).

## Positionality

### Definition and relevance to research

In order for the reader to assess the effect of the researcher’s identity on the research process and results, researchers must account for the identity they have chosen to adopt within a given research study, known as positionality (Savin-Baden & Major, 2013). Positionality is normally identified by locating the researcher’s position in relationship to three areas: the topic under investigation; the research participants; and the research design, context and process (Holmes, 2020). However, positionality is not only affected by the position the researcher themselves chooses to adopt but also by the way they are positioned by others (Arber, 2006). This includes study participants, gatekeepers and other collaborators in the research.

### Insider, outsider or in-betweener

Researcher positionality is commonly discussed in the literature as a clear distinction between insider (emic) and outsider (etic) perspectives (Huberman & Miles, 2002). Insiders are considered part of the community within which they are conducting research while outsiders are considered to be outside of the group they are studying. Some scholars warn against this binary distinction, arguing instead that insider and outsider perspectives are two ends of a positionality continuum along which researchers move back and forth during the research process in a dynamic, continuous way (Arber, 2006). Others have argued for the concept of an ‘in-betweener’ researcher, who identifies as neither entirely inside or outside (Chhabra, 2020; Milligan, 2014).

Conducting research as an insider, for example a paramedic conducting research on or with paramedics within their specific clinical setting, has the advantage that the researcher is already immersed in the organisation and has built up knowledge of the organisation (Brannick & Coghlan, 2007). Consequently, access to participants may be easier when interviewing other paramedics or people known to the researcher in a professional context (Chew-Graham et al., 2002). During data collection, participants may be less cautious or guarded than they would be with an outsider researcher, resulting in more genuine data (Chew-Graham et al., 2002).

However, prior knowledge can affect the way that the researcher is perceived, the information that participants provide and the analysis of those data (Chew-Graham et al., 2002). Participants may assume that the insider researcher sees things the way they do because of their common profession, or may even seek to impress or agree with the researcher based on their perceived connection (Chew-Graham et al., 2002).

The role duality experienced by clinicians and researchers who are acting as ‘double agents’ (Yanos & Ziedonis, 2006, p. 249) raises questions around power dynamics, as participants may be concerned about giving the right answers (Wiles et al., 2006). The researcher should continually reflect upon which position they are adopting throughout the research and how this may influence data collection and analysis. An illustrated example of this is provided in Table 2.

**Table 2.** Illustrated example – positionality.

I am an experienced paramedic undertaking a PhD on pre-hospital feedback. I have existing views and experience of pre-hospital feedback, namely I am passionate about the provision of feedback and believe it improves patient care. My clinical experience has guided the choice of my research topic and research question. When returning to clinical duties after an extended time off the road, I frequently asked staff at the receiving hospital for feedback on the outcomes of my patients. Locally, there were some formal mechanisms to support this, which I accessed, but I also often resorted to informally seeking feedback from doctors and nurses. I also actively sought feedback from colleagues who I worked with on my decision making and recall encouraging others to do the same. With time, I became frustrated with what I perceived to be inadequate provision of feedback to emergency ambulance staff and I pursued a PhD to explore this topic, with the aim to enhance pre-hospital feedback provision and thereby improve clinical decision-making and staff well-being. My clinical background has continued to shape the research I have undertaken as part of my PhD. Specifically, it has informed my qualitative interview topic guide, interview technique and data analysis, due to having an in-depth personal understanding of the contextual elements mentioned by participants. For quantitative aspects of my PhD, it has shaped my research questions and study design elements, such as selecting multiple short electronic diary entries – rather than one long survey or using a paper survey – as well as piloting my survey items with paramedic colleagues to ensure readability and understanding. My insider positionality meant I was familiar with stakeholders such as the National Ambulance Research Steering Group and key routes for dissemination such as the 999 EMS Research Forum and the College of Paramedics conferences. It has also allowed me to plan for wider impact beyond these obvious dissemination strategies, such as the development of a best practice guideline for pre-hospital feedback and co-design of a personal toolkit for front line clinicians on how to seek and use feedback in the pre-hospital setting. Without this insider knowledge, I may not have recognised the need for activities beyond conference presentations and journal articles to ensure that my research findings influence clinical practice.

## Reflexivity

### Purpose and process

Addressing the issues of researcher identity and positionality requires reflexivity throughout the planning and conducting of a research study, in order to produce a trustworthy and honest account (Probst, 2015). Reflexivity involves the researcher building on the recognised and clearly stated assumptions (i.e. identity and positionality), by questioning and addressing these assumptions using strategies pertaining to: the research topic; the research design, context and process; and the research participants.

Practical recommendations for strategies that our workshop attendees utilised to question and address their underlying assumptions were: researcher diaries; involving non-clinicians in the research project; pre-registration of the research study; respondent validation/member-checking; triangulation of data sources; and positionality statements. Other examples from the literature include using a social identity map for practising explicit positionality, debriefing with other researchers to work through problems, returning to the raw data, consulting the broader literature to situate findings in existing knowledge and practising collaborative reflexivity between team members to contribute to a multi-faceted understanding (Jacobson & Mustafa, 2019; Probst, 2015). Specifically for pre-doctoral or doctoral clinician-researchers conducting interviews, McNair et al. (2008) suggests having experienced research supervisors, arranging pilot interviews that include active feedback on interviewing style and being reflexive during interviews.

### Reflexivity versus reflective practice

Paramedics will be familiar with the concept of reflective practice; therefore, it is important to highlight how reflexivity differs from this. Reflective practice is an ongoing way of thinking, which involves the clinician looking inwards and reflecting on what they have learnt and what it means to them (Bolton & Delderfield, 2018). In contrast, reflexivity is about the researcher finding strategies to question their attitudes in an outward-looking perspective, while considering the implications of what they have learnt for the wider context they work with (Bolton & Delderfield, 2018; Mathers, 2019).

Although the strategies applied as part of reflexivity will vary according to the needs of the researcher and the research study, the principles of questioning and addressing assumptions remain the same. Rae and Green (2016) offer a useful model for supporting reflexivity in health services research, which influenced the lead author when devising the strategies to address reflexivity as part of their doctoral studies, depicted in Table 3.

**Table 3.** Illustrated example – reflexivity.

Systematic review	Utilised a researcher diary to note down decisions made during screening, data extraction and analysis Discussed decisions and preliminary analysis with the multidisciplinary research team
Qualitative interviews	Introduced myself as a paramedic, i.e. an insider Experienced supervisor provided feedback on interview technique Built-in capacity for respondent validation Topic guide was shaped by clinical experience, existing literature and multidisciplinary research team input Used existing theory during data analysis Highlighted divergent findings during analysis write-up
Quantitative studies	Protocol externally peer-reviewed Pre-registered, published and/or presented at conferences
Overall PhD	Continually exploring and developing clinical academic identity Positionality statement Sought wider input from stakeholders outside of the multidisciplinary research team

## Conclusions

Paramedics carrying out research in their immediate clinical setting and within their broader work environment need to have an appreciation and understanding of their identity, positionality and reflexivity. We have outlined how a researcher’s identity and positionality can influence all aspects of research, including the research question, study design, data collection and data analysis. We have discussed that the insider position of paramedics conducting research with paramedics or within their specific clinical setting has considerable benefits to participant access, understanding of data and dissemination, while highlighting the difficulties of role duality and power dynamics. Pre-hospital and out-of-hospital research is a relatively novel area of clinical research, which paramedics are well-placed to pursue, given their insider position. Throughout this article, we have provided examples of how the concepts of identity, positionality and reflexivity have been considered and applied by a research paramedic while conducting their PhD research studies within the paramedic community, to illustrate how they can be practically applied.

## Acknowledgements

The authors would like to acknowledge the vital contributions made by the attendees at the interactive workshop on research paramedic identity and positionality at the College of Paramedics Annual International Research Conference 2021. CW would like to thank Greg Whitley for his assistance in refining Figure 1, and acknowledge the support received from members of the Manchester Metropolitan University Research Methods Community of Practice led by Prof. Karen Sage and Dr. Gillian Janes, when CW presented this work in January 2022.

## Author contributions

CW conceived the idea for this manuscript and drafted the initial version under guidance and supervision from GJ and JW. CW and JW hosted the interactive workshop. All authors revised the manuscript and approved the final version to be published. CW acts as the guarantor for this article.

## Conflict of interest

CW and JW are on the editorial board of the *BPJ*.

## Ethics

Ethics approvals were not required for this manuscript as it is a methodological discussion article and does not constitute research according to the Health Research Authority Decision Tool. Ethics approvals were obtained for the PhD research studies referred to in this article as illustrative examples of identity, positionality and reflexivity.

## Funding

This research was funded by the National Institute for Health Research (NIHR) Yorkshire and Humber Patient Safety Translational Research Centre (YHPSTRC) and an NIHR Short Placement Award for Research Collaboration (SPARC) awarded to CW. The views expressed are those of the author(s) and not necessarily those of the NIHR or the Department of Health and Social Care. The funders had no role in study design, data collection and analysis, decision to publish or preparation of the manuscript.
